# Monitoring heparin therapy: stability of two different anti-Xa assays using blood samples collected in citrate-containing and CTAD tubes

**DOI:** 10.1186/s12959-023-00465-8

**Published:** 2023-02-20

**Authors:** Marion Gremillet, Laurie Talon, Aurélien Lebreton, Thomas Sinegre

**Affiliations:** 1 CHU Clermont-Ferrand, Service d’hématologie Biologique, 1 Place Lucie Et Raymond Aubrac, 63003 Clermont-Ferrand, France; 2 Université Clermont Auvergne, Unité de Nutrition Humaine, UMR INRAE/UCA 1019, Clermont-Ferrand, France

**Keywords:** Blood coagulation tests, Drug monitoring, Heparin, Haemostasis, Pre-analytical phase

## Abstract

**Background:**

Anti-factor Xa assays and activated partial thromboplastin time (aPTT) are mainly employed to monitor patients treated with heparins. According to the Clinical and Laboratory Standards Institute and the French Working Group on Haemostasis and Thrombosis, anti-factor Xa activity and aPTT should be tested within 2 h of blood sampling for unfractionated heparin (UFH) monitoring. However, discrepancies exist depending on the used reagents and collecting tubes. The study aim was to determine the stability of aPTT and anti-factor Xa measurements using blood samples collected in citrate-containing or citrate-theophylline-adenosine-dipyridamole (CTAD) tubes and stored for up to 6 h.

**Methods:**

Patients receiving UFH or low molecular weight heparin (LMWH) were enrolled; aPTT and anti-factor Xa activity were tested using two different analyser/reagent pairs (Stago and reagent without dextran sulfate; Siemens and reagent with dextran sulfate) after 1, 4 and 6 h of sample storage as whole blood or as plasma.

**Results:**

For UFH monitoring, comparable anti-factor Xa activity and aPTT results were obtained with both analyser/reagent pairs when samples were stored as whole blood before plasma isolation. With samples stored as plasma, anti-factor Xa activity and aPTT were not affected up to 6 h after sampling when using the Stago/no-dextran sulfate reagent pair. With the Siemens/dextran sulfate-containing reagent, aPTT was significantly altered after 4 h of storage. For LMWH monitoring, anti-factor Xa activity remained stable (whole blood and plasma) for at least 6 h. Results were comparable with citrate-containing and CTAD tubes.

**Conclusions:**

Anti-factor Xa activity in samples stored as whole blood or plasma was stable for up to 6 h, regardless of the reagent (with/without dextran sulfate)/collection tube. Conversely, aPTT was more variable because other plasma parameters can influence its measure and complicate the interpretation of its variations after 4 h.

**Supplementary Information:**

The online version contains supplementary material available at 10.1186/s12959-023-00465-8.

## Introduction

Heparins are the most used injectable anticoagulants. They increase the inhibitory activity of antithrombin [1], particularly on thrombin when using unfractionated heparin (UFH) and on activated factor X (Xa) when using low molecular weight heparin (LMWH). UFH is largely used in intensive care units due to its short half-life, compatibility with renal failure, and availability of a reversal agent [2]. However, monitoring UFH treatment is a major issue because of the high anticoagulant variability among patients [3], the narrow therapeutic window, and the risk of thrombosis or bleeding following under- or over-dosing [4]. UFH can be monitored by measuring the anti-factor Xa activity or activated partial thromboplastin time (aPTT) [5]. Conversely, patients receiving LMWH do not need routine laboratory monitoring because fixed weight-adjusted doses are administered. However, anti-factor Xa activity testing is recommended in some cases, such as obesity, renal failure, extreme age, and pregnancy [6].

When samples collected in citrate-containing tubes are stored as whole blood (WB) before plasma isolation for UFH monitoring, stability is relatively short: 1 h according to the Clinical and Laboratory Standards Institute (CLSI) guidelines [7], and 2 h according to the French Working Group on Haemostasis and Thrombosis (GFHT) [8]. However, recent studies have shown acceptable stability of aPTT and anti-factor Xa activity assays when samples were stored as WB for 4 h [9, 10]. According to the French guidelines, samples can be used up to 4 h when centrifuged within 1 h of collection and then the plasma is stored at room temperature [8]. For LMWH monitoring, the GFHT recommends to store WB samples for 6 h at most [8]. However, this recommendation is based on two studies on LMWH stability with discordant results [11, 12].

Delays in sample shipping to the testing laboratory site are increasing due to the centralisation of laboratories and are a real logistical issue. Citrate, Theophylline, Adenosine, Dipyridamole (CTAD) tubes give a better stability [7, 8], but they do not seem to be a viable alternative due to their limited availability, high cost, and impossibility to explore platelet functions. In addition, due to the lack of anti-factor Xa activity assay standardisation for the different marketed reagents, sample stability must be controlled for each assay [13]. An important difference among the existing reagents is the presence or absence of dextran sulfate that reduces the impact of heparin-binding proteins, particularly platelet factor 4 (PF4) [14, 15].

Thus, the aim of this prospective study was to determine WB and plasma stability for aPTT and anti-factor Xa measurements over 6 h using samples collected in citrate-containing or CTAD tubes and two main reagent/analyser pairs, one with and one without dextran sulfate.

## Materials and methods

### Blood sampling

Adult patients receiving UFH or LMWH were enrolled at Clermont-Ferrand university hospital (France) between May 2020 and February 2021. Blood was drawn from an antecubital vein with a light tourniquet and a 21G needle and collected, after discarding the first few millilitres, into a tube containing 0.109 M citrate (Vacutainer, Becton Dickinson, Le Pont-de-Claix, France), 9:1 v/v, or a tube containing CTAD (0.109 M, Becton Dickinson). All samples were transported by a pneumatic tube system to the laboratory. Experiments were performed in accordance with the French authorities and approved by the Clermont-Ferrand university hospital ethical committee (CPP Sud-Est VI, ref. AU765).

### Stability study design

WB stability was evaluated by equally dividing the initial tube into three aliquots that were stored at room temperature for 1 h (T1WB), 4 h (T4WB) and 6 h (T6WB) respectively, before centrifugation at 2500 g at room temperature for 15 min for plasma isolation and analysis within 10 min. For plasma stability testing, the initial tube was centrifuged (2500 g at room temperature for 15 min) within 1 h after collection. Platelet-poor plasma was then decanted and stored at room temperature until analysis after 1 h (T1P), 4 h (T4P) and 6 h (T6P) of storage.

### Anti-factor Xa activity and activated partial thromboplastin time

Less than 10 min after centrifugation of the WB samples stored for different amounts of time (T1WB, T4WB, T6WB), aPTT and anti-factor Xa activity were tested with two different reagent/analyser couples: STA-PTT-A and STA-Liquid Anti-Xa (without dextran sulfate) used on a STA-R Max (Diagnostica Stago, Asnières-sur-Seine, France) and Actin FS and Innovance Heparin (with dextran sulfate) used on a CS2100 (Siemens, Saint-Denis, France). The same analyses were performed also with the plasma samples at T1P, T4P, T6P.

### Statistical analysis

Statistical analyses were performed with the Prism software, version 9.0 (GraphPad Software, USA). Results were expressed as medians (min–max) in IU/mL for anti-factor Xa activity and as ratios for aPTT.

Tests were two-sided, with a type I error set at α = 0.05. Groups were compared using the ANOVA or Friedman test when the ANOVA conditions were not met (normality and homoscedasticity verified with the Shapiro test). Bland–Altman plots were used to evaluate the mean bias among test results obtained at 1 h, 4 h and 6 h.

For UFH monitoring, the concordance of test results (within or outside the therapeutic range) after 6 h/4 h and 1 h was evaluated using the kappa score Ks (agreement: excellent if Ks > 0.8; good if 0.6 < Ks < 0.8; moderate if 0.4 < Ks < 0.6; poor if Ks < 0.4). Results were considered within the therapeutic range if they were between 0.30 and 0.70 IU/mL for anti-factor Xa activity and between 1.5 and 3 for the aPTT ratio.

## Results

### Stability of samples collected in citrate-containing tubes

#### Stability of WB samples from patients treated with UFH

**Fig. 1:**
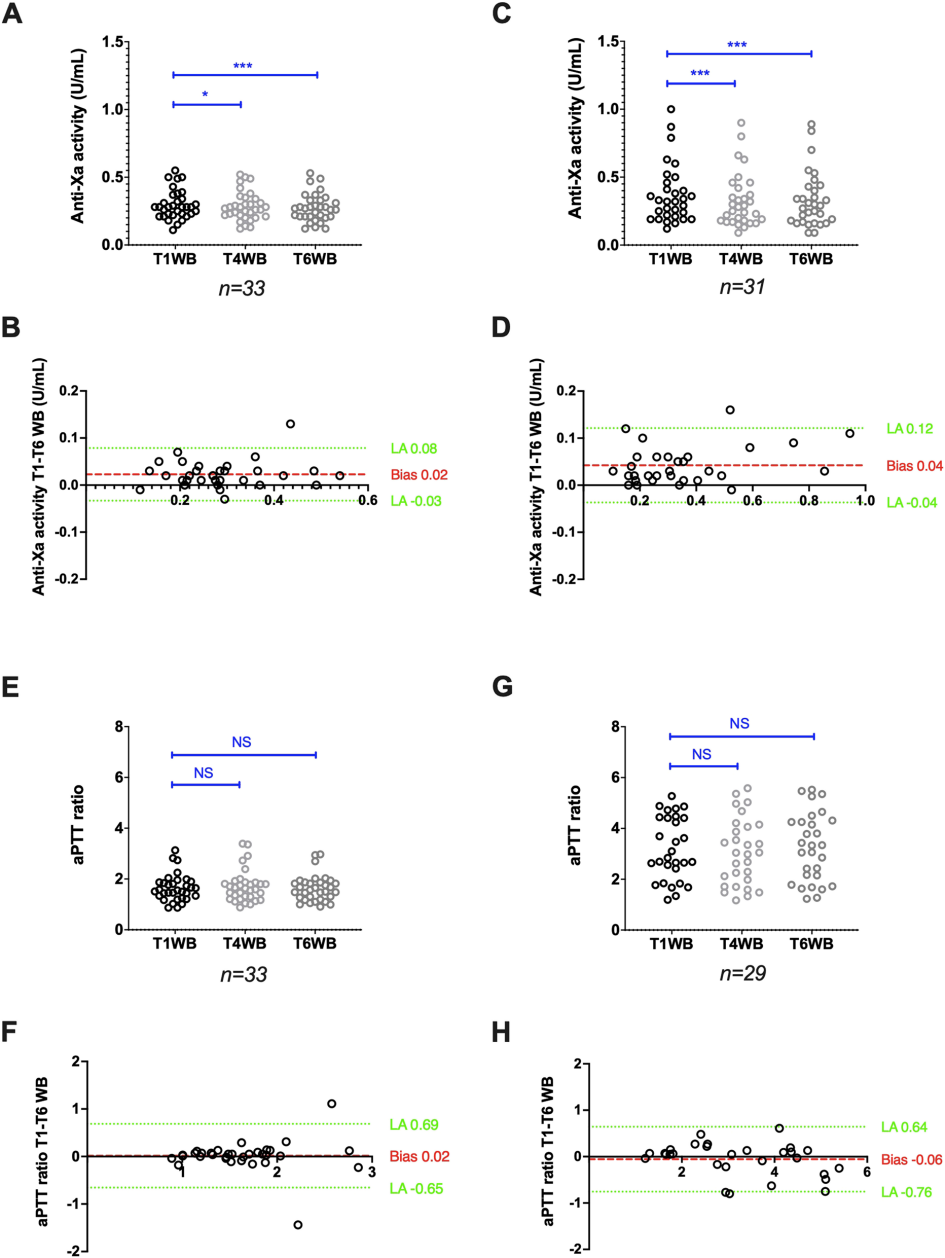
Stability of citrated whole blood samples from patients treated with UFH. Anti-factor Xa activity scatter plots (A and C) and Bland–Altman plots (B and D), and aPTT ratio scatter plots (E and G) and Bland–Altman plots (F and H) after analysis with the Siemens and Stago reagent/analyser combination, respectively. Results are plotted as the difference (y axis) in anti-factor Xa activity or aPTT ratio between the analysis after 6 h (T6WB), 4 h (T4WB) and 1 h (T1WB) of storage at room temperature. *P < 0.05, **P < 0.01, ***P < 0.001, NS: Not significant (ANOVA or non-parametric Friedman test)

Anti-factor Xa activities (IU/mL), measured with the Siemens reagent/analyser combination (*n* = 33), decreased from 0.28 (0.11–0.55) at T1WB to 0.27 (0.12–0.52) at T4WB (*p* < 0.05) and to 0.27 (0.12–0.53) at T6WB (*p* < 0.001), but the bias between T1WB and T6WB was limited to -0.02 IU/mL (Fig. 1A,B). For the Stago reagent/analyser combination (*n* = 31), anti-factor Xa activities decreased from 0.32 (0.12–1.00) at T1WB to 0.28 (0.09–0.90) at T4WB (*p* < 0.001) and to 0.29 (0.09–0.89) at T6WB (*p* < 0.001), and the bias between T1WB and T6WB was limited to -0.04 IU/mL (Fig. 1C,D). The agreement between T1WB and T6WB was good for the Siemens (kappa score Ks = 0.73) and excellent for the Stago (Ks = 0.80) reagent/analyser pair.

The aPTT ratio, measured with the Siemens reagent/analyser combination (*n* = 33), remained stable: 1.55 (0.87–3.13) at T1WB and 1.57 (0.91–2.97) at T6WB (*p* = 0.07). The bias between T1WB and T6WB was -0.02 (Fig. 1E,F). With the Stago reagent/analyser combination (*n* = 29), an upward, but not significant, trend was observed for the aPTT ratio: 2.84 (1.19–5.27) at T1WB and 3.33 (1.23–5.52) at T6WB (*p* = 0.40). The bias between T1WB and T6WB was 0.06 (Fig. 1G,H). The aPTT agreement between T1WB and T6WB was good for both the Siemens (Ks = 0.64) and Stago (Ks = 0.79) reagent/analyser combinations.

### Stability of plasma samples from patients treated with UFH

**Fig. 2:**
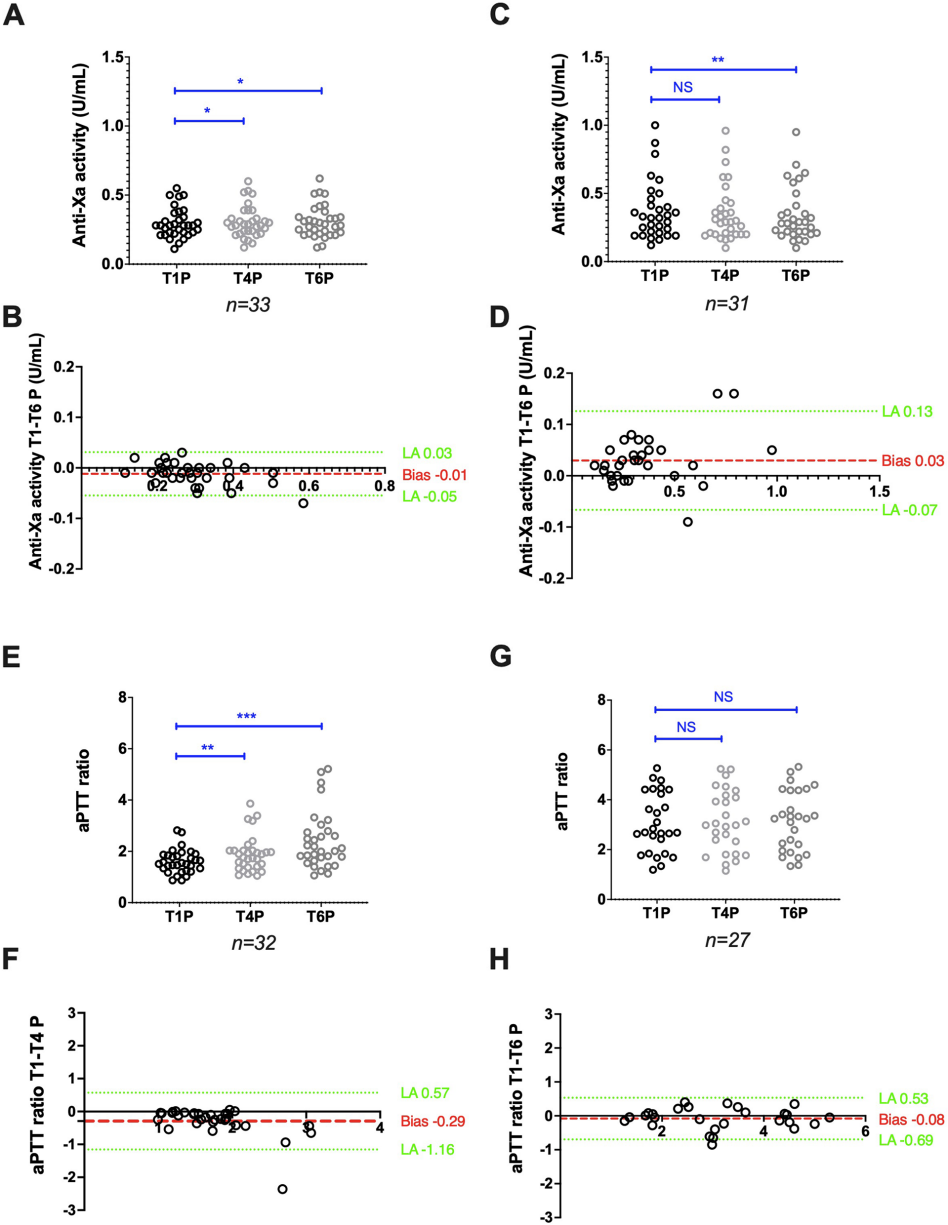
Stability of citrated plasma samples from patients treated with UFH. Anti-factor Xa activity scatter plots (A and C) and Bland–Altman plots (B and D), and aPTT ratio scatter plots (E and G) and Bland–Altman plots (F and H) obtained using the Siemens and Stago analyser/reagent pairs, respectively. Results are plotted as the difference (y axis) in anti-factor Xa activity or aPTT ratio between the analysis after 6 h (T6P) or 4 h (T4P) and 1 h (T1P) of storage at room temperature. *P < 0.05, **P < 0.01, ***P < 0.001, NS: Not significant (ANOVA or non-parametric Friedman test)

Anti-factor Xa activities measured with the Siemens reagent/analyser combination (*n* = 33) increased from 0.28 (0.11–0.55) at T1P to 0.29 (0.12–0.60) at T4P (*p* < 0.05) and to 0.29 IU/mL (0.12–0.62) at T6P (*p* < 0.05). The bias between T1P and T6P was limited to 0.01 IU/mL (Fig. 2A,B). Using the Stago reagent/analyser combination (*n* = 31), anti-factor Xa activities decreased from 0.32 (0.12–1.00) at T1P to 0.28 (0.10–0.95) at T6P (*p* < 0.01), but remained stable between T1P and T4P [0.29 (0.10–0.96); *p* = 0.11)]. The bias between T1P and T6P was -0.03 IU/mL (Fig. 2C,D). The agreement between T1P and T6P was good for both the Siemens (Ks = 0.76) and Stago (Ks = 0.67) reagent/analyser combinations.

The aPTT ratio, assessed with the Siemens reagent/analyser pair (*n* = 32), increased from 1.53 (0.87–2.82) at T1P to 1.83 (1.05–3.86) at T4P (*p* < 0.01). The bias between T1P and T4P was quite high: 0.29 (Fig. 2E,F). Using the Stago reagent/analyser combination, the aPTT ratio (*n* = 27) remained stable over time: 2.69 (1.19–5.27) at T1P and 3.24 (1.34–5.32) at T6P (*p* = 0.47). The bias between T1P and T6P was 0.08 (Fig. 2G,H). The aPTT ratio agreement was moderate for the Siemens (Ks = 0.51, between T1P and T4P) and good for the Stago reagent/analyser combination (Ks = 0.70, between T1P and T6P).

### Stability of samples from patients treated with LMWH

**Fig. 3:**
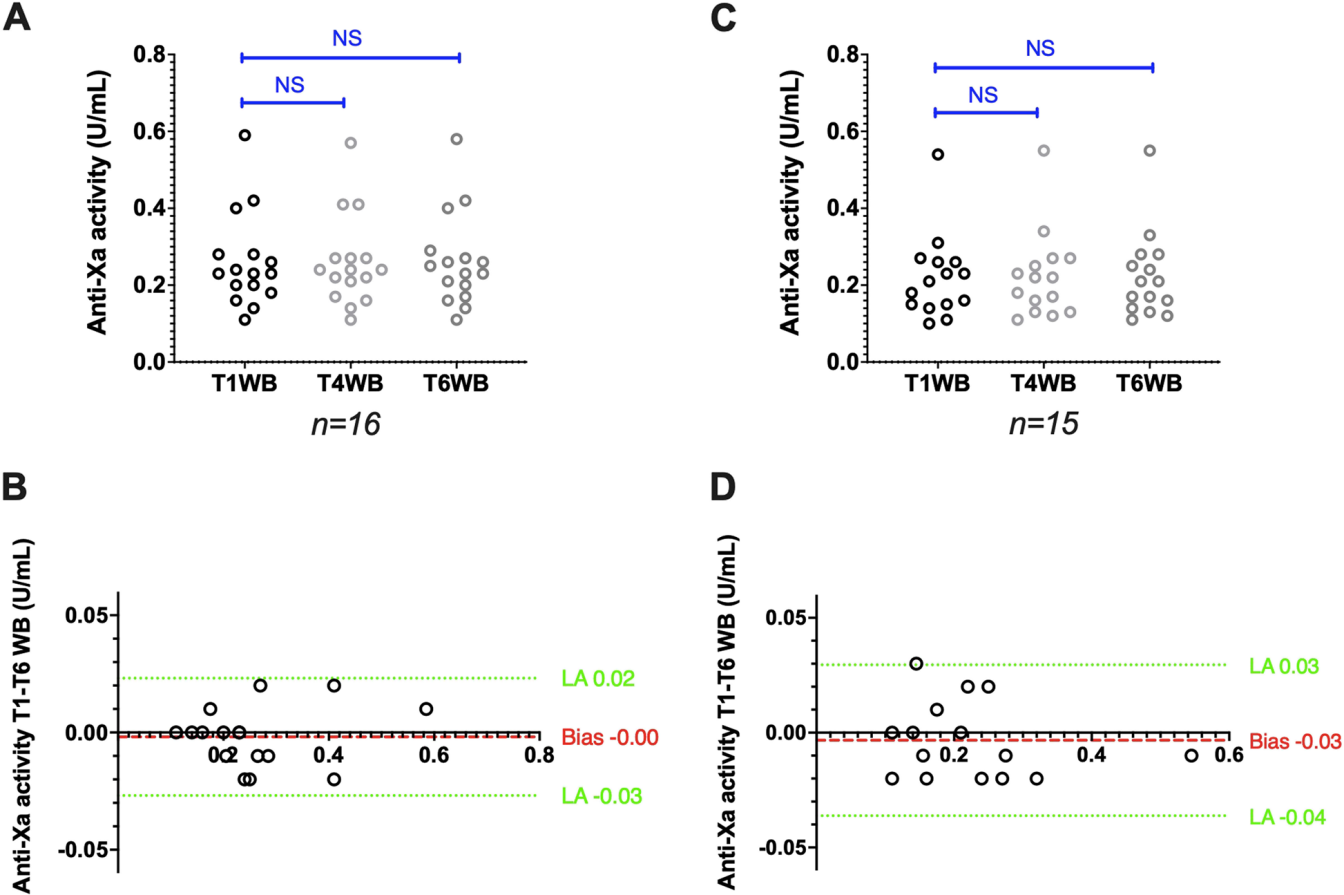
Stability of citrated whole blood samples from patients treated with LMWH. Anti-factor Xa activity scatter plots (A and C) and Bland–Altman plots (B and D) obtained with the Siemens and Stago analyser/reagent pairs, respectively. Results are plotted as the difference (y axis) in anti-factor Xa activity between the analysis after 6 h (T6WB), 4 h (T4WB) and 1 h (T1WB) of storage at room temperature. *P < 0.05, **P < 0.01, ***P < 0.001, NS: Not significant (ANOVA or non-parametric Friedman test)

In sample stored as WB, anti-factor Xa activities measured using the Siemens analyser/reagent pair (*n* = 16) remained unchanged: 0.23 (0.11–0.59) at T1WB and 0.24 (0.11–0.58) at T6WB (*p* > 0.99). The bias was 0.00 IU/mL (Fig. 3A,B). Anti-factor Xa activities measured using the Stago analyser/reagent pair (*n* = 15) also were stable: 0.21 (0.10–0.54) at T1WB and 0.21 (0.11–0.55) at T6WB (*p* = 0.82). The bias was 0.03 IU/mL (Fig. 3C,D).

**Fig. 4:**
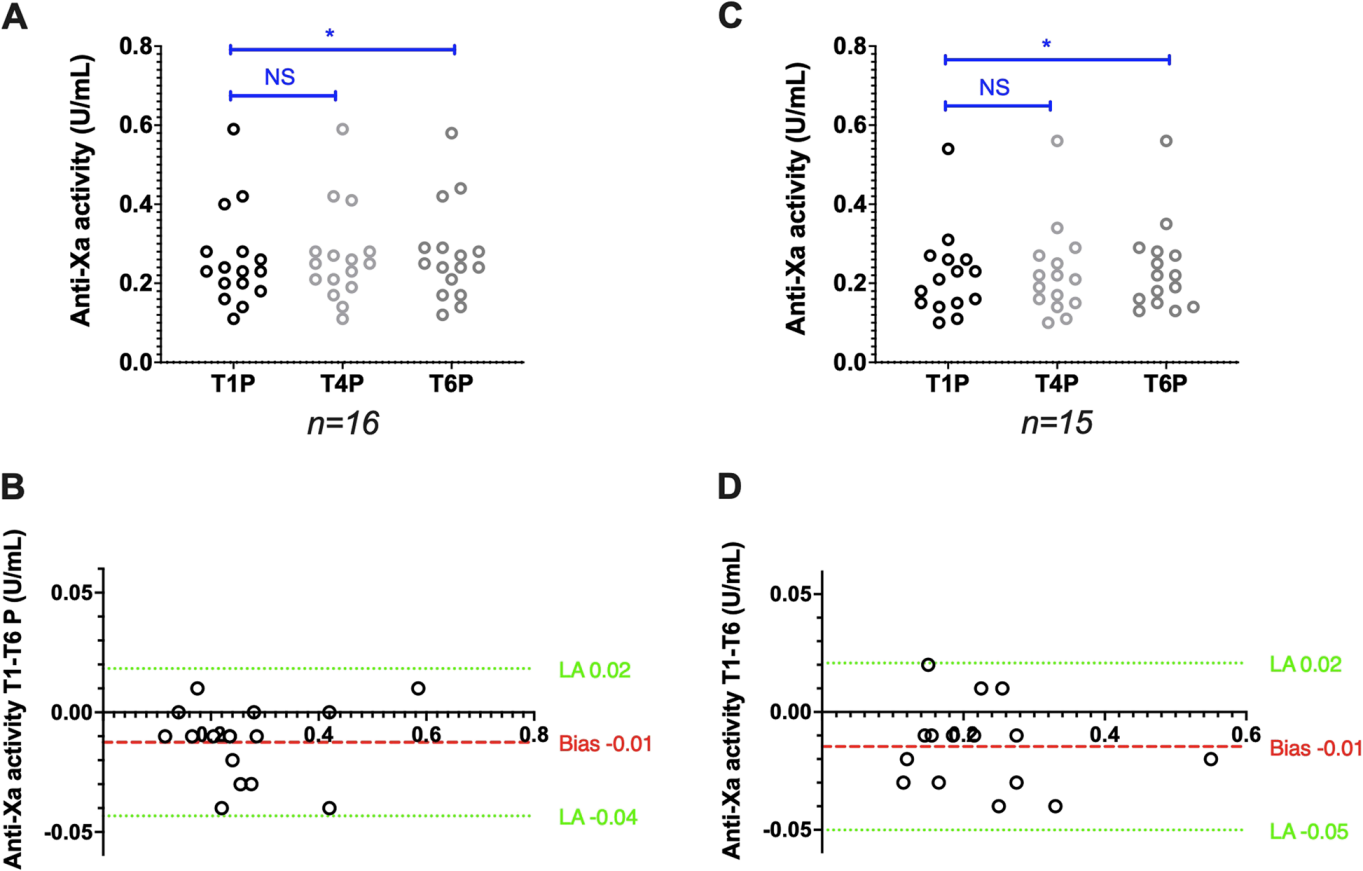
Stability of citrated plasma samples from patients treated with LMWH. Anti-factor Xa activity scatter plots (A and C) and Bland–Altman plots (B and D) analysed with the Siemens and Stago analyser/reagent pairs, respectively. Results are plotted as the difference (y axis) in anti-factor Xa activity between the analysis after 6 h (T6P), 4 h (T4P) and 1 h (T1P) of storage at room temperature. *P < 0.05, **P < 0.01, ***P < 0.001, NS: Not significant (ANOVA or non-parametric Friedman test)

In samples stored as plasma, anti-factor Xa activities measured using the Siemens analyser/reagent pair (*n* = 16) remained stable between T1P and T4P [0.23 (0.11–0.59) vs 0.25 (0.11–0.59); *p* = 0.23] and increased between T1P and T6P [0.23 (0.11–0.59) vs 0.25 (0.12–0.58); *p* < 0.05]. The bias between T1P and T6P was limited to 0.01 IU/mL (Fig. 4A,B). With the Stago analyser/reagent pair (*n* = 15), anti-factor Xa activities were stable between T1P and T4P [0.21 (0.10–0.54) vs 0.21 (0.10–0.56); *p* = 0.43] and increased between T1P and T6P [0.21 (0.10–0.54) vs 0.22 (0.13–0.56); *p* < 0.05]. The bias between T1P and T6P was limited to 0.01 IU/mL (Fig. 4C,D).

### Stability of blood samples collected in CTAD tubes

#### Stability of WB samples from patients treated with UFH

**Fig. 5:**
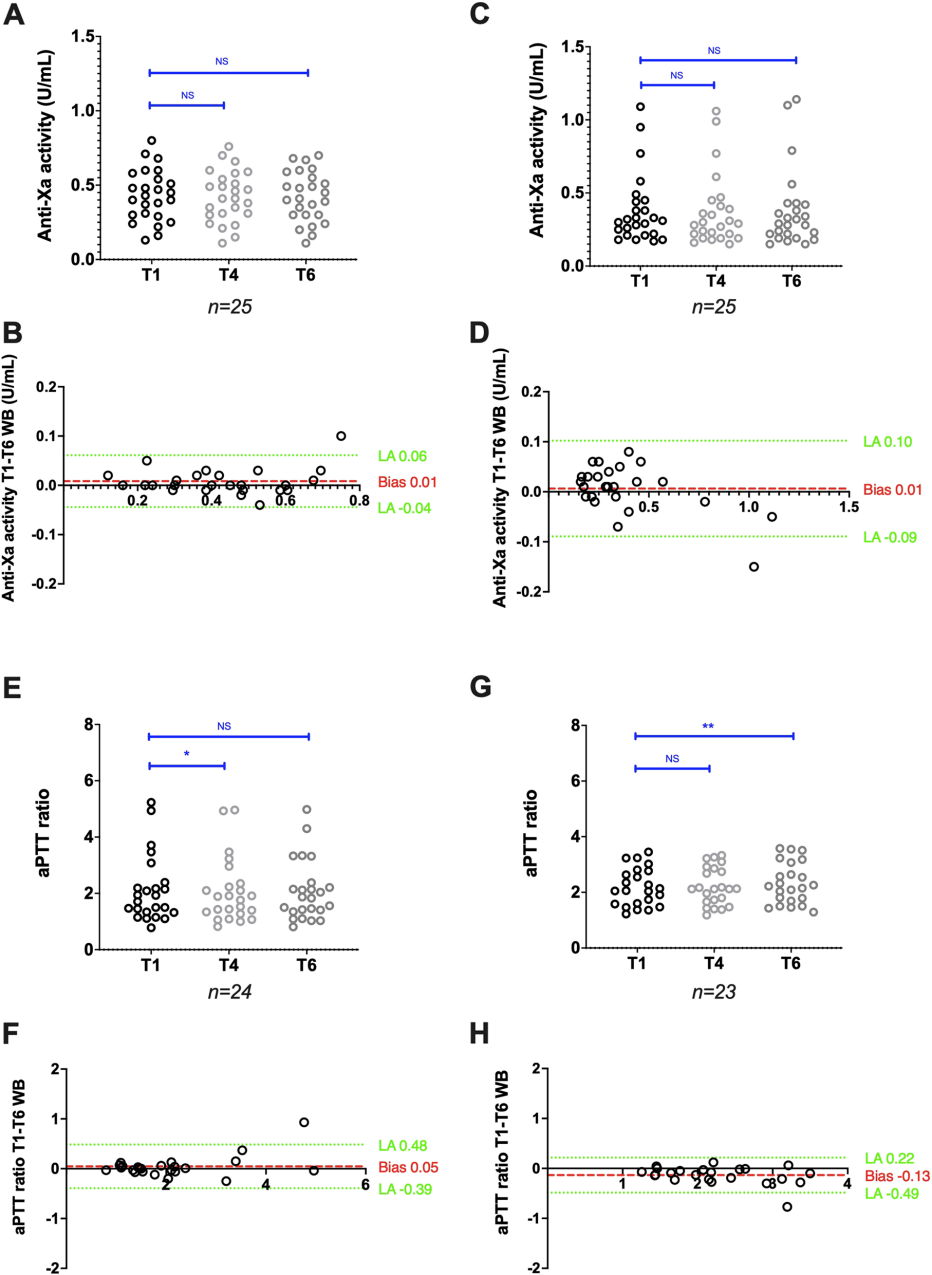
Stability of CTAD whole blood samples from patients treated with UFH. Anti-factor Xa activity scatter plots (A and C), Bland–Altman plots (B and D), and aPTT ratio scatter plots (E and G), Bland–Altman plots (F and H) after analysis with the Siemens and Stago reagent/analyser combination, respectively. Results are plotted as the difference (y axis) in anti-factor Xa activity or aPTT ratio between the analysis after 6 h (T6WB), 4 h (T4WB) and 1 h (T1WB) of storage at room temperature. *P < 0.05, **P < 0.01, ***P < 0.001, NS: Not significant (ANOVA or non-parametric Friedman test)

Anti-factor Xa activities (IU/mL), measured with the Siemens reagent/analyser combination (*n* = 25), remained stable: 0.43 (0.13–0.80) at T1WB and 0.41 (0.11–0.70) at T6WB (*p* = 0.25). The bias between T1WB and T6WB was -0.01 IU/mL (Fig. 5A,B). With the Stago reagent/analyser combination (*n* = 25), anti-factor Xa activities also remained unchanged: 0.31 (0.17–1.09) at T1WB and 0.29 (0.15–1.14) at T6WB (*p* = 0.20). The bias between T1WB and T6WB was -0.01 IU/mL (Fig. 5C,D). The agreement between T1WB and T6WB was good for the Siemens (Ks = 0.69) and excellent for the Stago (Ks = 0.83) reagent/analyser pair.

The aPTT ratio, measured with the Siemens reagent/analyser combination (*n* = 24), remained stable: 1.82 (0.78–5.23) at T1WB and 1.85 (0.81–4.98) at T6WB (*p* > 0.99). The bias between T1WB and T6WB was -0.05 (Fig. 5E,F). With the Stago reagent/analyser combination (*n* = 23), the aPTT ratio increased from 2.06 (1.22–3.45) at T1WB to 2.22 (1.29–3.58) at T6WB (*p* < 0.01). The bias between T1WB and T6WB was 0.13 (Fig. 5G,H). The aPTT agreement between T1WB and T6WB was excellent for the Siemens (Ks = 1) and good for the Stago (Ks = 0.63) reagent/analyser combination.

### Stability of plasma samples from patients treated with UFH

**Fig. 6:**
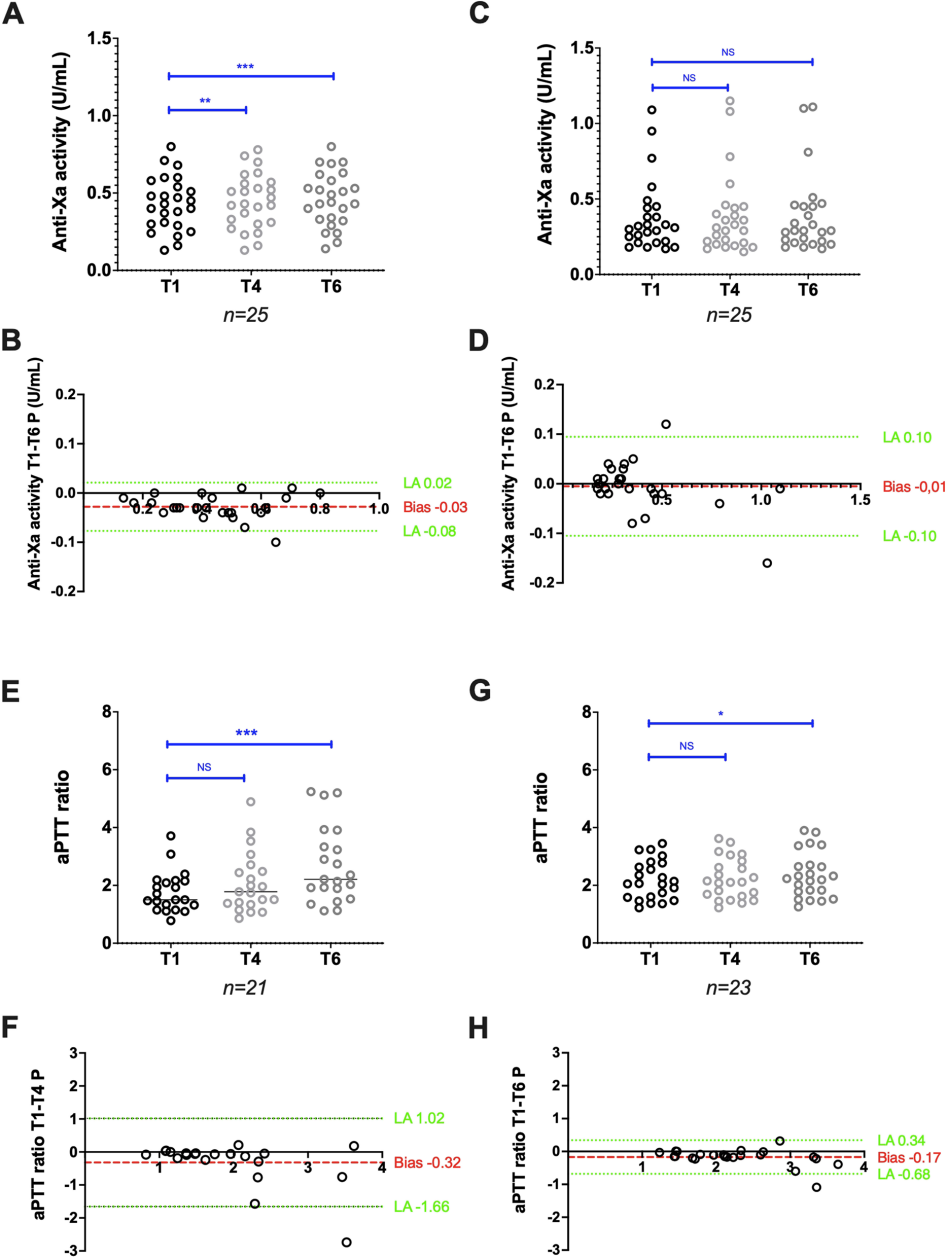
Stability of CTAD plasma samples from patients treated with UFH. Anti-factor Xa activity scatter plots (A and C) and Bland–Altman plots (B and D), and aPTT ratio scatter plots (E and G) and Bland–Altman plots (F and H) obtained using the Siemens and Stago analyser/reagent pair, respectively. Results are plotted as the difference (y axis) in anti-factor Xa activity or aPTT ratio between the analysis after 6 h (T6P) or 4 h (T4P) and 1 h (T1P) of storage at room temperature. *P < 0.05, **P < 0.01, ***P < 0.001, NS: Not significant (ANOVA or non-parametric Friedman test)

Anti-factor Xa activities measured with the Siemens reagent/analyser combination (*n* = 25) increased from 0.43 (0.13–0.80) at T1P to 0.43 (0.13–0.78) at T4P (*p* < 0.01) and to 0.44 (0.14–0.80) at T6P (*p* < 0.001). However, the bias between T1P and T6P was limited to 0.03 IU/mL (Fig. 6A,B). Using the Stago reagent/analyser combination (*n* = 25), anti-factor Xa activities remained stable: 0.31 (0.17–1.09) at T1P and 0.29 (0.17–1.11) at T6P (*p* > 0.99). The bias between T1P and T6P was 0.01 IU/mL (Fig. 6C,D). The agreement between T1P and T6P was excellent for the Siemens (Ks = 0.80) and good for the Stago (Ks = 0.75) reagent/analyser combination.

The aPTT ratio, assessed with the Siemens reagent/analyser pair (*n* = 21), increased from 1.50 (0.78–3.71) at T1P to 2.21 (1.12–5.24) at T4P, but the difference was not statically significant (*p* = 0.09). The bias between T1P and T4P was quite high: 0.32 (Fig. 6E,F). Using the Stago reagent/analyser combination, the aPTT ratio (*n* = 23) increased from 2.06 (1.22–3.45) at T1P to 2.23 (1.25–3.90) at T6P (*p* < 0.05). The bias between T1P and T6P was 0.17 (Fig. 6G,H). The aPTT ratio agreement between time points was moderate for both the Siemens (Ks = 0.57 between T1P and T4P) and the Stago (Ks = 0.53 between T1P and T6P) reagent/analyser combinations.

### Stability of samples from patients treated with LMWH

**Fig. 7:**
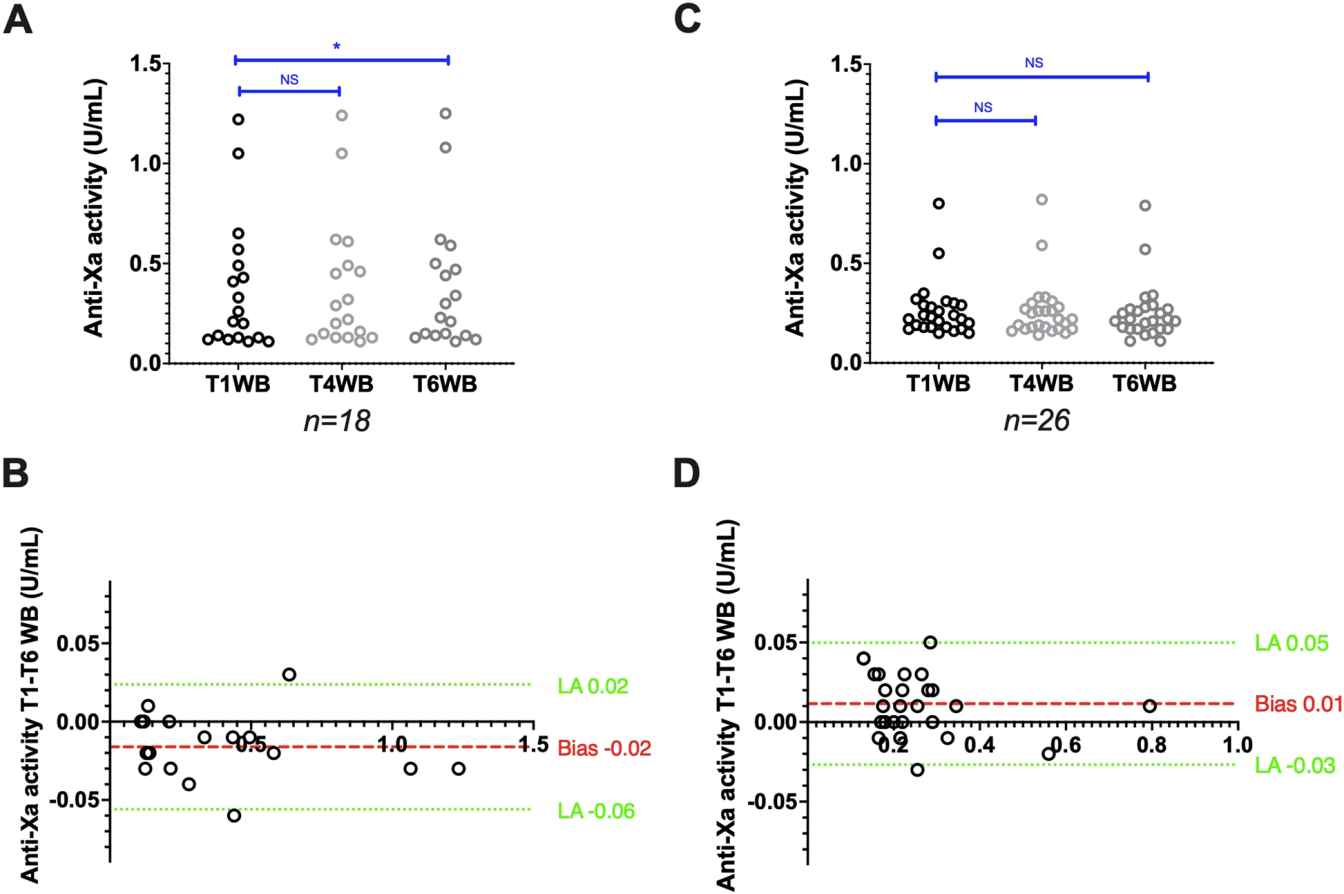
Stability of CTAD whole blood samples from patients treated by LMWH. Anti-factor Xa activity scatter plots (A and C) and Bland–Altman plots (B and D) obtained with the Siemens and Stago analyser/reagent pairs, respectively. Results are plotted as the difference (y axis) in anti-factor Xa activity between the analysis after 6 h (T6WB), 4 h (T4WB) and 1 h (T1WB) of storage at room temperature. *P < 0.05, **P < 0.01, ***P < 0.001, NS: Not significant (ANOVA or non-parametric Friedman test)

In samples stored as WB, anti-factor Xa activities measured using the Siemens analyser/reagent pair (*n* = 18) were stable between T1P and T4P [0.24 (0.11–1.22) vs 0.26 (0.11–1.24); *p* = 0.20] and increased between T1P and T6P [0.24 (0.11–1.22) vs 0.27 (0.11–1.25); *p* < 0.05]. However, the bias between T1P and T6P was limited to 0.02 IU/mL (Fig. 7A,B). With the Stago analyser/reagent pair (*n* = 26), anti-factor Xa activities remained stable between T1P and T4P [0.23 (0.15–0.80) vs 0.22 (0.14–0.82); *p* > 0.99] and between T1P and T6P [0.22 (0.11–0.79); *p* = 0.18]. The bias between T1P and T6P was -0.01 IU/mL (Fig. 7C,D).

**Fig. 8:**
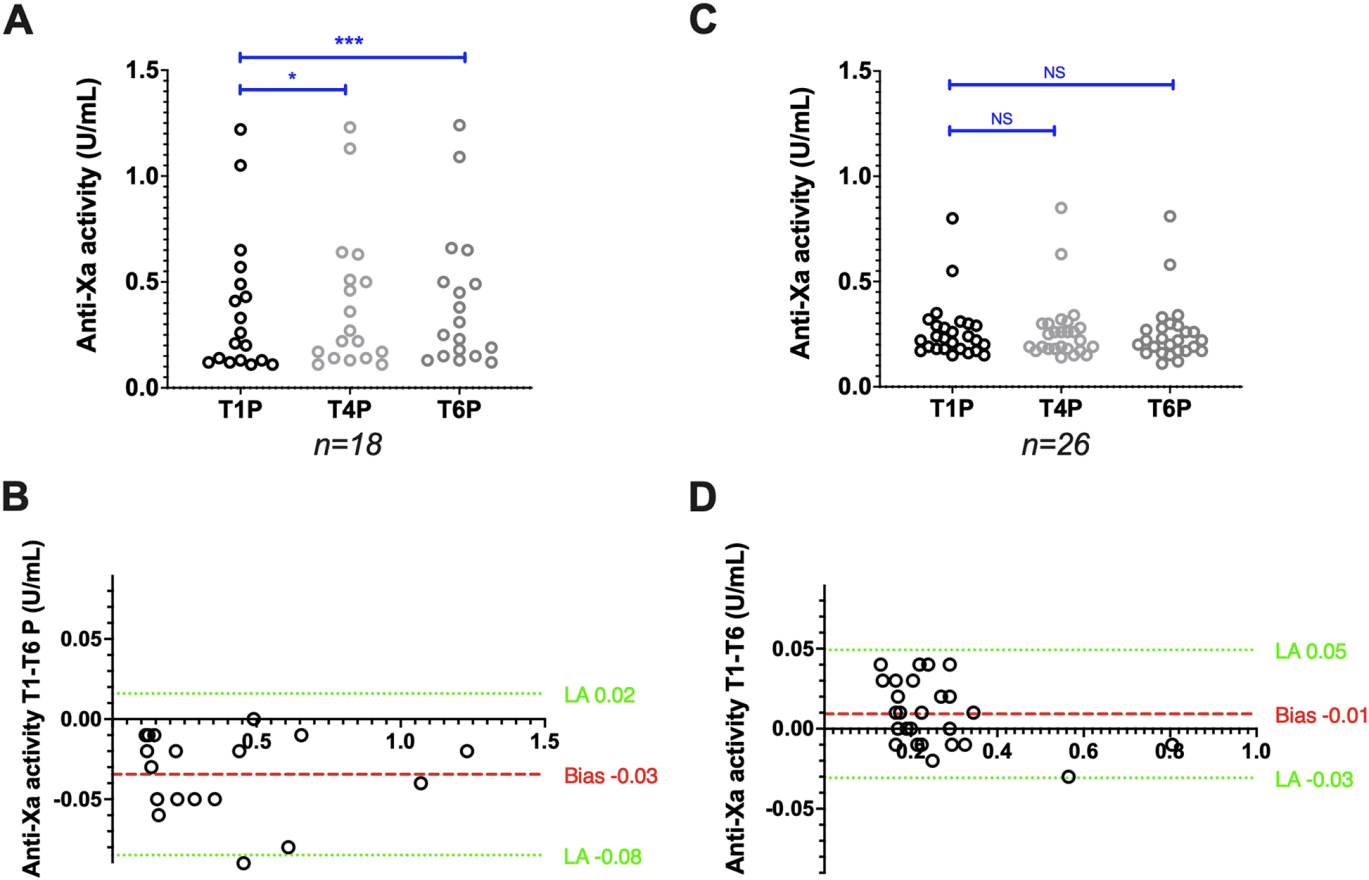
Stability of CTAD plasma samples from patients treated with LMWH. Anti-factor Xa activity scatter plots (A and C) and Bland–Altman plots (B and D) analysed with the Siemens and Stago analyser/reagent pairs, respectively. Results are plotted as the difference (y axis) in anti-factor Xa activity between the analysis after 6 h (T6P), 4 h (T4P)and 1 h (T1P) of storage at room temperature. *P < 0.05, **P < 0.01, ***P < 0.001, NS: Not significant (ANOVA or non-parametric Friedman test)

In samples stored as plasma, anti-factor Xa activities measured using the Siemens analyser/reagent pair (*n* = 18) increased between T1P and T4P [0.24 (0.11–1.22) vs 0.25 (0.11–1.23); *p* < 0.05] and between T1P and T6P [0.24 (0.11–1.22) vs 0.28 (0.12–1.24); *p* < 0.001]. The bias between T1P and T6P was 0.03 IU/mL (Fig. 8A,B). With the Stago analyser/reagent pair (*n* = 26), anti-factor Xa activities were stable between T1P and T4P [0.23 (0.15–0.80) vs 0.24 (0.14–0.85); *p* > 0.99] and between T1P and T6P [0.22 (0.11–0.81); *p* = 0.64]. The bias between T1P and T6P was 0.01 IU/mL (Fig. 8C,D).

## Discussion

Here, we found a global stability of 6 h for the different combinations of reagent/analyser and storage conditions. The measured variations over time are consistent with the performance of the technique. Indeed, our repeatability and reproducibility data varied from 2 and 5% and from 7 and 10%, respectively, between the lowest and highest measured values. Based on previous works, it is recommended to use citrate-containing WB stored for less than 2 h for UFH monitoring [16–18]. Two more recent studies concluded that anti-factor Xa activity measurement could be extended to 4 h [9, 10]. The present findings suggest that both anti-factor Xa activity and aPTT could be measured (Siemens and Stago) up to 6 h after sampling without any clinical impact. 

Our study also showed that for samples collected in citrate-containing tubes and stored as plasma, anti-factor Xa activity measurement could be extended from < 4 h [8, 17, 18] to 6 h without any significant clinical impact, for both the Stago and Siemens analyser/reagent pairs. The presence of dextran sulfate in the Siemens (but not Stago) reagent should reduce the influence of heparin antagonists, especially PF4 [14] that neutralises anti-factor Xa activity [15]. Therefore, it should theoretically avoid underestimating heparin concentration or even lead to an overestimation of UFH activity [19]. However, this hypothesis was rejected because anti-factor Xa activity remained stable with both combinations. A summary of the literature findings on the stability of samples collected in citrate-containing tubes is presented in Supplemental material Table 1.

On the other hand, for aPTT measurement, our data differed in function of the reagent. Plasma stability could be extended up to 6 h without any clinical impact with the Stago, but not with the Siemens analyser/reagent pair. aPTT is a global test sensitive to heparin, but also to other coagulation factors of the intrinsic pathway, particularly factor VIII [20]. Factor VIII stability in plasma is limited to 4 h before decreasing importantly (-20%), which could lead to an aPTT increase [8, 21, 22]. Therefore, the lower stability of the Siemens reagent could be explained by the fact that Actin FS is more sensitive to factor deficiencies, especially factors VIII, IX and XI, compared with PPT-A [23].

Heparin inactivation in blood is mainly attributed to PF4 that is released by platelet α- granules [15]. As CTAD prevents platelet aggregation and release, the use of CTAD tubes should reduce heparin inactivation during blood centrifugation and storage [24]. Consequently, blood storage in CTAD tubes might represent the best pre-analytical condition for reliable heparin treatment monitoring by aPTT, compared with citrate-containing tubes [25]. French [8] and international guidelines [7] recommend to use CTAD tubes to improve aPTT stability for heparin monitoring, although only few studies evaluated the stability over time of blood samples collected in CTAD tubes.

Our results showed no difference in stability between blood samples collected in CTAD and citrate-containing tubes for both aPTT and anti-factor Xa activity and all studied combinations (storage as WB or plasma; Siemens or Stago reagent/analyser pair). The recommendations on stability of blood samples collected in CTAD tubes are based on very few studies. One study has evaluated the stability at 4 h and our data supports the current recommendations, including for reagents containing dextran sulfate [9, 18]. A summary of the literature findings on the stability of samples collected in CTAD tubes is presented in Supplemental material Table 2.

These data suggest that the main decrease in aPTT occurs during blood sampling rather than during storage at room temperature for few hours. Therefore, aPPT measurement using blood samples collected in CTAD tubes represents more closely the true in vivo levels of UFH at venipuncture time; however, CTAD does not improve stability during storage compared with citrate-containing tubes [18]. Due to its size and chemical structure, LMWH binds less to PF4 [15]. This minimises the risk of underestimating the anti-factor Xa activity [8]. Our data indicate that for LMWH monitoring, blood samples collected in citrate and CTAD tubes are stable for at least 6 h (WB and plasma). This is in line with previous recommendations [8] based on a single study that used a dextran sulfate-containing reagent [11]. Our study also showed that anti-factor Xa activity is stable for 6 h even when using a reagent without dextran sulfate (Stago). A summary of the literature data on LMWH stability in samples collected in citrate-containing tubes is in Supplemental material Table 3.

## Conclusion

Anti-Xa activity testing has become the reference assay for heparin monitoring because it is influenced by fewer factors compared with aPTT and is used in intensive care units. Anti-factor Xa activity was stable for 6 h when using blood samples collected in citrate-containing or CTAD tubes and stored as WB or plasma, regardless of the reagent (with/without dextran sulfate) and analyser used.

The aPTT ratio was more variable because other plasma parameters, such as intrinsic pathway factors and particularly factor VIII, can affect its measurement and complicate the interpretation of its variations over time.

## Supplementary Information

**Additional file 1:** **Table 1.** Stability of samples collected in citrate-containing tubes for UFH monitoring. **Table 2.** Stability of samples collected in CTAD tubes for UFH monitoring. **Table 3.** Stability of blood samples collected in citrate-containing tubes for LMWH monitoring.

## Data Availability

Available at CHU Clermont-Ferrand France from the corresponding author.

## Abbreviations

aPTT: Activated partial thromboplastin time
AXA: Anti-activated factor X activity
CLSI: Clinical and Laboratory Standards Institute
CTAD: Citrate, Theophylline, Adenosine, Dipyridamole
GFHT: French Working Group on Haemostasis and Thrombosis
Ks: Kappa score
LMWH: Low molecular weight heparin
PF4: Platelet factor 4
UFH: Unfractionated heparin
WB: Whole blood
Xa: Activated factor X
