# Supplementary material for: Monitoring heparin therapy: stability of two different anti-Xa assays using blood samples collected in citrate-containing and CTAD tubes
Source: Thromb J. 2023 Feb 20;21:21. doi: 10.1186/s12959-023-00465-8 (PMC9942401; doi:10.1186/s12959-023-00465-8)
Supplement: Supplementary file 1 — Additional file 1: Table 1. Stability of samples collected in citrate-containing tubes for UFH monitoring. Table 2. Stability of samples collected in CTAD tubes for UFH monitoring. Table 3. Stability of blood samples collected in citrate-containing tubes for LMWH monitoring. [file 12959_2023_465_MOESM1_ESM.docx]

**Supplemental material**

|  | **Parameters** | **Reagent** | **Analyser** | **Stability** | |
| --- | --- | --- | --- | --- | --- |
|  |  |  |  | **WB** | **Plasma** |
| **Adcock et al. 1998 [17]** | aPTT | Actin FS (Dade) | MLA model 900/1000 (Medical laboratory automation) | 1 hour | / |
| **Heil et al. 1998 [21]** | aPTT | ? | Fibrintimer A (Behringwerke) | / | < 8 hours* |
| **Awad et al. 2005 [16]** | aPTT | Dade Behring Test Kit | ? | Immediately | / |
| **Ray et al. 2008 [18]** | aPTT | Platelin LS (Biomerieux) | STA-R (Stago) | 100 minutes | / |
|  | AXA | Rotachrom heparin assay (Stago) |  | 100 minutes | / |
| **CLSI 2008 [7]** | aPTT | / | / | 1 hour | 4 hours** |
|  | AXA | / | / | 4 hours | 4 hours |
| **GFHT 2017 [8]** | aPTT/AXA | / | / | 2 hours | 4 hours** |
| **Billoir et al. 2019 [9]** | aPTT | STA R PTT-A (Diagnostica Stago) | STA R Max (Diagnostica Stago) | 4 hours | / |
|  | AXA | STA R-Liquid anti-Xa (Diagnostica Stago) without dextran sulfate |  | 4 hours | / |
| **Toulon et al. 2020 [10]** | aPTT | HemosIL SynthASil reagent (Instrumental Laboratory, IL) | ACL Top 700 CTS (IL) | 1-2 hours | / |
|  | AXA | Biophen Heparin LRT (Hyphen Biomed) with dextran sulfate  HemosIL Liquid Heparin (IL) with dextran sulfate |  | 4 hours | / |
| **Our study** | aPTT | STA PTT-A (Stago)  Actin FS (Siemens) | STA-R (Stago)  CS2100 (Siemens) | 6 hours | 6 hours (Stago)  <4 hours (Siemens) |
|  | AXA | STA Liquid Anti-Xa (Stago) without dextran sulfate  Innovance Heparin (Siemens) with dextran sulfate |  | 6 hours | 6 hours |

**Table 1: Stability of samples collected in citrate-containing tubes for UFH monitoring**

According to the current recommendations, UFH should be measured in blood samples collected in citrate-containing tubes within 4 hours after sampling. This has been confirmed by recent studies using reagents adapted to the current market. Our data show that this interval can be extended up to 6 hours, particularly for measuring anti-factor Xa activity (in the presence or not of dextran sulfate).

AXA: anti-facor Xa activity; WB: whole blood; *centrifugation as soon as possible; **centrifugation within 1 hour of sampling

|  | **Parameters** | **Reagent** | **Analyser** | **Stability** | |
| --- | --- | --- | --- | --- | --- |
|  |  |  |  | **WB** | **Plasma** |
| **Ray et al. 2008 [18]** | aPTT | Platelin LS (Biomerieux) | STA-R (Stago) | 100 minutes | / |
|  | AXA | Rotachrom heparin assay (Stago) |  | 100 minutes | / |
| **CLSI 2008 [7]** | aPTT | / | / | > citrate | / |
| **GFHT 2017 [8]** | aPTT/AXA | / | / | 6 hours | 6 hours |
| **Billoir et al. 2019 [9]** | aPTT | STA R PTT-A (Diagnostica Stago) | STA R Max (Diagnostica Stago) | 4 hours | / |
|  | AXA | STA R-Liquid anti-Xa (Diagnostica Stago) without dextran sulfate |  | 4 hours | / |
| **Our study** | aPTT | STA PTT-A (Stago)  Actin FS (Siemens) | STA-R (Stago)  CS2100 (Siemens) | 6 hours | 4 hours (Stago)  <4 hours (Siemens) |
|  | AXA | STA Liquid Anti-Xa (Stago) without dextran sulfate  Innovance Heparin (Siemens) with dextran sulfate |  | 6 hours | 6 hours |

**Table 2: Stability of samples collected in CTAD tubes for UFH monitoring**

The recommendations on stability of blood samples collected in CTAD tubes are based on very few studies. Since then, a study has tested stability at 4 hours and our data supports the current recommendations, including for reagents containing dextran sulfate.

AXA: anti-factor Xa activity; UFH: unfractionned heparin; WB, whole blood.

|  | **Parameters** | **Reagent** | **Analyser** | **Stability** | |
| --- | --- | --- | --- | --- | --- |
|  |  |  |  | **WB** | **Plasma** |
| **Rojnuckarin et al. 2010 [12]** | AXA | Berichrom heparin (Dade Behring) with dextran sulfate | CA-500 (Sysmex) | / | <24 hours |
| **Birri et al. 2011 [11]** | AXA | Biophen Heparin 6 (Hyphen Biomed) with dextran sulfate | BCS-XP (Siemens) | 6 hours | 24 hours |
| **GFHT 2017 [8]** | AXA | / | / | 6 hours | 6 hours |
| **Our study** | AXA | STA Liquid Anti-Xa (Stago) without dextran sulfate  Innovance Heparin (Siemens) with dextran sulfate | STA-R (Stago)  CS2100 (Siemens) | 6 hours | 6 hours |

**Table 3: Stability of blood samples collected in citrate-containing tubes for LMWH monitoring.**

The stability of anti-factor Xa activity measurement for LMWH monitoring has been rarely studied. Our data confirm the current recommendations, based essentially on the publication by Birri et al., of a stability of 6 hours. These recommendations can be used also for reagents containing dextran sulfate.

AXA: anti-factor Xa activity; LMWH: low molecular weight heparin; UFH: unfractionated heparin; WB: whole blood
